# Differential Entropy Preserves Variational Information of Near-Infrared Spectroscopy Time Series Associated With Working Memory

**DOI:** 10.3389/fninf.2018.00033

**Published:** 2018-06-05

**Authors:** Soheil Keshmiri, Hidenubo Sumioka, Ryuji Yamazaki, Hiroshi Ishiguro

**Affiliations:** ^1^Hiroshi Ishiguro Laboratories, Advanced Telecommunications Research Institute International, Kyoto, Japan; ^2^School of Social Sciences, Waseda University, Tokyo, Japan; ^3^Graduate School of Engineering Science, Osaka University, Suita, Japan

**Keywords:** near-infrared spectroscopy, differential entropy, NIRS time series feature extraction, brain activity decoding, working memory

## Abstract

Neuroscience research shows a growing interest in the application of Near-Infrared Spectroscopy (NIRS) in analysis and decoding of the brain activity of human subjects. Given the correlation that is observed between the Blood Oxygen Dependent Level (BOLD) responses that are exhibited by the time series data of functional Magnetic Resonance Imaging (fMRI) and the hemoglobin oxy/deoxy-genation that is captured by NIRS, linear models play a central role in these applications. This, in turn, results in adaptation of the feature extraction strategies that are well-suited for discretization of data that exhibit a high degree of linearity, namely, slope and the mean as well as their combination, to summarize the informational contents of the NIRS time series. In this article, we demonstrate that these features are inefficient in capturing the variational information of NIRS data, limiting the reliability and the adequacy of the conclusion on their results. Alternatively, we propose the linear estimate of differential entropy of these time series as a natural representation of such information. We provide evidence for our claim through comparative analysis of the application of these features on NIRS data pertinent to several working memory tasks as well as naturalistic conversational stimuli.

## 1. Introduction

Recent years witness a growing interest in Near-Infrared Spectroscopy (Ferrari and Quaresima, 2012; Dix et al., 2013) as a promising tool for analysis of the brain activity of human subjects. Cui et al. (2010a) define NIRS as a technology for functional brain imaging based on hemodynamic signals from the cortex. The operational principle of NIRS devices is based on recording of the optical absorption of light over time to estimate the functionally evoked changes in cerebral oxy/deoxy-hemoglobin concentrations that result from local cerebral vascular and oxygen metabolic effects during the brain activity (Huppert et al., 2009). This technology is successfully applied in a variety of research areas, ranging from monitoring of the cerebral and myocardial oxygen sufficiency (Jobsis, 1977) and effect of aging on working memory (Vermeij et al., 2012) to decoding of vigilance (Bogler et al., 2014), complexity analysis of the neural activity of the children with attention-deficit/hyperactivity disorder (ADHD) (Gu et al., 2017), behavioral differences between the genders (Baker et al., 2016), functioning of frontal cortex (Ozawa et al., 2014; Perlman et al., 2015), as well as determination of the level of difficulty of the mental tasks (Verner et al., 2013), to name a few.

Utilization of NIRS for monitoring the brain activity becomes more attractive, considering the non-invasive operational setup of NIRS-related devices. These devices are easy to use with portable, light-weighted headsets that are comparatively more immune to body movement (Dieler et al., 2012) while preserving unrestrictiveness and accessibility along with compact experimental setting (Moriai-Izawaa et al., 2012). These advantages provide a tremendous opportunity for their adaptation in naturalistic experiments through elimination of the requirement for confining the human subject to fMRI chamber (Naseer and Hong, 2015; Shin et al., 2016). It is worthy of note that although NIRS has a weaker signal-to-noise ratio (SNR) in comparison with fMRI (Gagon et al., 2011), research suggests that the hemodynamic responses that it captures in the form of blood oxy/deoxy-genation hemoglobin highly correlates with the BOLD in fMRI (Strangman et al., 2002; Steinbrink et al., 2006; Cui et al., 2011). Moreover, this correlation is unaffected by the nature of cognitive tasks (Strangman et al., 2002; Steinbrink et al., 2006; Cui et al., 2011). Such spatio-temporal similarities between BOLD and blood oxygenation (i.e., Δ oxy-Hb) are further supported by a number of research findings (Okamoto et al., 2004; Strangman et al., 2002; Toronov et al., 2007).

An important characteristic that is attributable to NIRS and fMRI time series is the underlying linear property of the hemodynamic responses that are measured by these devices (Dale and Buckner, 1997; Henson et al., 2001; Henson, 2003; Penny et al., 2005), particularly in prefrontal cortex (PFC) (Braver et al., 1997; Mitchell et al., 2008; Fishbum et al., 2014). Friston et al. (1994a) first investigate this linear property of hemodynamic responses, leading to formalization of General Linear Model (GLM) (Friston et al., 2011). Later, Schroeter et al. (2004) and Plichta et al. (2007) independently validate the applicability of GLM to NIRS time series. Along the same axis, Kamran and Hong (2014) argue that NIRS time series is a linear combination of various components, ranging from dynamical characteristics of the oxy/deoxy-hemoglobin (HbO/HbR) changes in a specific brain region to the baseline effect (Gusnard and Raichle, 2001), thereby further extending the results in (Friston et al., 1994a; Plichta et al., 2007; Friston et al., 2011). The overall acceptance of such an underlying linear dynamics is strengthened, considering the adaptation of GLM and its variation in fNIRS-fMRI comparative studies (e.g., Steinbrink et al., 2006; Sato et al., 2013, 2016) as well as its inclusion in major f/NIRS statistical analysis and modeling toolboxes (Koh et al., 2007; Huppert et al., 2009; Strangmann, 2009; Ye et al., 2009; Fekete et al., 2011). Tak and Ye (2013) present a comprehensive review of the statistical analysis and modeling of f/NIRS time series.

These results, in turn, help explain the choice of feature extraction methodologies that are well-suited for discretization of data with a high degree of linearity (i.e., linear changes in hemodynamics in response to a given stimulus), namely, the slope (Herff et al., 2014), the mean (Fazli et al., 2012; Khan et al., 2014) as well as their combination^1^ (Naseer and Hong, 2013; Shin et al., 2016) to summarize the information content of NIRS time series. This claim is further supported by Cui et al. (2010b) whose comparative analysis suggest that the slope (i.e., a linear correlate) of the NIRS data forms a significantly informative feature in contrast to various feature spaces. However, averaging-based feature extraction strategies are ill-suited for capturing the essential information pertinent to brain responses to stimuli (Spiers and Maguire, 2007; Erceg-Hurn and Mirosevich, 2008; Hasson and Honey, 2012; Wilcox, 2012, 2017; Wehbe et al., 2014; Liu et al., 2017; Rousselet et al., 2017). In fact, Ben-Yakov et al. (2012) show the shortcoming of the averaging-based methods for detecting responses that are highly context dependent, resulting in worsening the effective signal-to-noise ratio. Moreover, Haynes and Rees (2007) claim that averaging of the brain activity loses its functional state at any given point in time. Rousselet et al. (2017) present a comprehensive survey and analysis on this matter.

On the other hand, research suggests a direct correspondence between variational behavior of the brain activity and its information content (Miller, 2001; Friston, 2010; Bastos et al., 2018; Lundqvist et al., 2018; Wutz et al., 2018). For instance, Miller (2001) argues that there is a direct correspondence between the “amount of information” and variance since “anything that increases the variance also increases the amount of information” (Miller, 2001). Along the same direction, Eden and Kramer Fano (1947) note the utility of Fano factor^2^ in characterization of neural spiking. Similarly, Cohen et al. (2017) consider the ability to identify meaningful variation in data pertinent to brain activation as an indicator of an effective analysis approach. Accordingly, Lundqvist et al. (2018) show that effect of working memory tasks is significantly captured by the variance of the information content of neural activation, thereby suggesting changes in variational information of spike rate to best represent the burst of brain activity in response to WM tasks. These results, collectively, suggest that the variation in time series of brain activity summarizes the cognitive load that is undertaken by the brain in processing a given stimulus.

These findings are in line with the concept of entropy in information theory (Cover and Thomas, 2006), as originally formulated by Shannon (1948). It comes as no surprise that entropy in its various formulations (Zanin et al., 2012; Gao et al., 2015; Xiong et al., 2017) is utilized exhaustively for analysis of the complexity and information content of biological signals (Costa et al., 2002; Lungarella and Sporns, 2006; Sengupta et al., 2013). In fact, its application expands over a wide spectrum, ranging from analysis of the complexity of heartbeats (Richman and Moorman, 2000; Bian et al., 2012) and state of anesthesia (Silva et al., 2010) to physiological complexity of aging and disease (Goldberger et al., 2002b), detection of epileptic seizure (Srinivasan and Eswaran, 2007) and investigation of vigilance and emotional states (Zhang and Lu, 2015). In addition, there exists a number of excellent and comprehensive entropy-based toolboxes for analysis of such time series signals (Ince et al., 2009; Lindner et al., 2011; Lizier, 2014).

In particular, linear estimate of differential entropy (Xiong et al., 2017) is used in study and analysis of a number of neurophysiological signals such as cardiovascular control mechanism (Porta et al., 2015) and cardiorespiratory dynamics (Faes et al., 2015). Shi et al. (2013) suggest the applicability of differential entropy (DE) i.e., the entropy of a continuous random variable (Cover and Thomas, 2006) in analysis of electroencephalography (EEG) time series. Furthermore, Keshmiri et al. (2017) show that DE significantly improves the classification accuracy of NIRS time series pertinent to cognitive load in prefrontal cortex during working memory task in comparison with a number of feature extraction and classification strategies. These results suggest the utility of DE to the solution concept of decoding of physiological and neurophysiological signals. However, lack of a theoretical evidence in its level of association for capturing the dynamical information of such time series data is apparent. In other words, there is a paucity of research in realization of the rationale underlying the shortcomings of averaging-based feature extraction strategies and, subsequently, the utility of adaptation of information-theoretic and variational-based approaches in calculating the summary statistics of brain activity.

In this article, we address these shortcomings through a systematic investigation of the mathematical foundation of the degree of correspondence between DE and these time series. In line with this perspective, we argue that averaging-based feature spaces are inefficient in representing the information content of the NIRS time series of the brain activity of human subjects. In doing so, we adapt the viewpoint of Miller (2001), thereby synonymously interpreting the amount of information conveyed by a NIRS time series as its variation, other than its overall trend or expectation. We further validate our claim by demonstrating the insensitivity of averaging-based feature spaces to such variational information, implicit in NIRS time series. Subsequently, we demonstrate that the linear estimate of differential entropy (Xiong et al., 2017) of these series help resolve this shortcoming.

Our contributions are twofold. Firstly, we present the mathematical bases for the shortcomings of the averaging-based feature spaces. Secondly, we prove that efficiency of the linear estimate of differential entropy of the time series of brain activity is due to its functional correspondence with the underlying spiking rate of neural activity. This is in line with the results in the literature, implying the correspondence between brain regional activation and the increase of the blood flow (Gusnard and Raichle, 2001). Additionally, we show the effectiveness of DE through examination of the feature extraction of the time series of the brain activity of human subjects, performing several working memory (WM) tasks. These WM tasks include Listening Span Test (LST), N-Back, Stroop, and Mental Arithmetic (MA). Moreover, we examine the utility of DE for capturing the information content of NIRS time series associated with more naturalistic scenarios using data pertinent to conversational tasks.

Our results suggest the potential that utilization of the linear estimate of the differential entropy of NIRS time series data can provide to the solution concept of the analysis of the brain activity of human subjects in response to working memory tasks whose variational intensities are nontrivial.

## 2. Materials and methods

### 2.1. Investigation of the feature spaces

As we presented in section 1, the central role of linear models in NIRS-based applications result in adaptation of the feature extraction strategies that are well-suited for discretization of data with a high degree of linearity, namely, the slope, the mean, and their combination to summarize the informational contents of the NIRS time series. In this section, we demonstrate the shortcomings of such feature spaces. Subsequently, we propose the use of linear estimate of DE of these time series as a natural choice for extracting the information content of these series.

It is apparent that slope is optimum if the data of a given time series is collinear and monotonic in its order. This is a substantial limiting factor since most physical system, including the brain, have their limits defined by their power limitation which corresponds to a limit on the variance of their outcome (Stone, 2015). In fact, Miller (2001) considers the amount of information, conceptually, synonymous with variance. Moreover, Pearl (1988) associates the expected cost of the best estimate of a given random variable to its variance. Furthermore, its significance is evident in a tremendous amount of research, dedicated to identifying the meaningful variation in the time series of the brain activity (Cohen et al., 2017). Given these remarks, it is plausible to interpret the efficiency of an adapted feature as its ability to capture the amount of variation in the data that it is applied on. We formalize the shortcoming of slope as a feature extraction strategy in capturing such a variational information of NIRS time series through following Proposition.

Proposition 2.1. *Slope is inefficient in capturing the variational information of the given time series*.

*Proof*. Slope, 𝕞, of a time series, *X*, is a measure of its linear trend with respect to the deviation of the observations from the fitted line that best minimizes these deviations. As such, the variance of the slope represents the estimate of the variational information of the observations, given this fitted line, i.e., Devore (2009)

(1) $$\begin{array}{l} \begin{array}{c} s_{𝕞}^{2}=\frac{\sum_{i=1}^{N}\left(x_{i}-\bar{x}\right)\left(t_{i}-\bar{t}\right)}{\sum_{i=1}^{N}{\left(t_{i}-\bar{t}\right)}^{2}}\\ =\frac{\sum_{i=1}^{N}{\left(x_{i}-\hat{x_{i}}\right)}^{2}}{\left(N-1\right)\sum_{i=1}^{N}{\left(t_{i}-\bar{t}\right)}^{2}} \end{array} \end{array}$$

where $s_{𝕞}^{2}$ is the variance of the slope of the sample time series data *x*_*i*_ ∈ *X* with *t*_*i*_ ∈ *T* being its corresponding independent variable (e.g., time stamps associated with the sample data and/or their respective indices). ||*X*|| = *N* = ||*T*||, $\bar{x}$, and $\bar{t}$ are their cardinality and sample means, respectively. Furthermore, $\frac{\overset{N}{\underset{i=1}{\sum }}{\left(x_{i}-\hat{x_{i}}\right)}^{2}}{N-1}$ is the estimate of the variation of the observations from the fitted line and *N*−1 is the degree-of-freedom. $\hat{x_{i}}$ indicates the vertical projection of the *i*th observation from the fitted line (i.e., its deviation). It is apparent that:

(2) $$\begin{array}{l} \overset{N}{\underset{i=1}{\sum }}{\left(x_{i}-\hat{x}\right)}^{2}\leq \overset{N}{\underset{i=1}{\sum }}{\left(x_{i}-\bar{x}\right)}^{2} \end{array}$$

Furthermore, for a monotonically increasing independent variable (e.g., time and/or indices of data point in time series), we have:

(3) $$\begin{array}{l} \left(N-1\right)\overset{N}{\underset{i=1}{\sum }}{\left(t_{i}-\bar{t}\right)}^{2}\geq \left(N-1\right)\Rightarrow \frac{1}{\left(N-1\right)\sum_{i=1}^{N}{\left(t_{i}-\bar{t_{i}}\right)}^{2}}\leq \frac{1}{N-1} \end{array}$$

Given the Equations (2) and (3), we have:

(4) $$\begin{array}{l} \begin{array}{c} \frac{\sum_{i=1}^{N}{\left(x_{i}-\hat{x_{i}}\right)}^{2}}{\left(N-1\right)\sum_{i=1}^{N}{\left(t_{i}-\bar{t}\right)}^{2}}\leq \frac{1}{N-1}\overset{N}{\underset{i=1}{\sum }}{\left(x_{i}-\bar{x}\right)}^{2}\\ \Rightarrow s_{𝕞}^{2}\leq s_{X}^{2} \end{array} \end{array}$$

where $s_{X}^{2}$ is the sample variance.

       ■

A customary practice in NIRS analysis is the transformation of data to ensure the reduction of the effect of the overall brain activity that is unrelated to the events of interests. These include subtraction/division of data with resting data (Fazli et al., 2012; Verner et al., 2013) to cancel the effects such as default mode (Gusnard and Raichle, 2001; Fransson, 2006; Fox and Raichle, 2007) of the brain, z-normalization (Bogler et al., 2014) i.e., $\frac{x-\mu_{X}}{\sigma_{X}},\;\forall x\in X$, for NIRS time series *X* with mean and standard deviation μ_*X*_ and σ_*X*_, and scaling within [0…1] interval (Hong et al., 2015) i.e., $\frac{x-X_{min}}{X_{max}-X_{min}},\;\forall x\in X$, with *X*_*min*_ and *X*_*max*_ indicating the minimum and maximum values in *X*. In fact, these steps are necessary to ensure reduction of undesirable effect such as biasing influence of outliers and/or residual effect of any previous cognitive load due to some unrelated mental activity. In other words, these steps help preserve the information gain from appropriate part of signal, given the adapted cognitive task. We utilize the concept of mutual information (Cover and Thomas, 2006; Stone, 2015) and its invariance under such transformations (Kinney and Atwal, 2014) to provide further evidence on inefficiency of slope as a feature extraction strategy for NIRS time series in the following Proposition.

Proposition 2.2. *Information gain based on the slope of a NIRS time series X under z-normalization and scaling is unwarranted*.

*Proof*. Let *X* be the original NIRS time series data and *y* its transformation such that:

(5) $$\begin{array}{l} y=g\left(X\right) \end{array}$$

where *g*(*X*) is of the forms $\frac{x-\mu_{X}}{\sigma_{X}}$ or $\frac{x-X_{min}}{X_{max}-X_{min}},\;\forall x\in X$ where μ_*X*_, σ_*X*_, *X*_*min*_, and *X*_*max*_ are mean, standard deviation, minimum, and maximum values of *X*, respectively. The entropy of *y* is (Stone, 2015):

(6) $$\begin{array}{l} H\left(y\right)=H\left(X\right)+E\left[\log|\frac{\partial g}{\partial X}|\right] \end{array}$$

The mutual information between *X* and *y* is:

(7) $$\begin{array}{l} \begin{array}{c} I\left(X,y\right)=H\left(X\right)-H\left(X|y\right)\\ =H\left(y\right)-H\left(y|X\right)\\ =H\left(X\right)+E\left[\log|\frac{\partial g}{\partial X}|\right]-H\left(y|X\right) \end{array} \end{array}$$

This implies that:

(8) $$\begin{array}{l} \begin{array}{c} H\left(X|y\right)=H\left(y|X\right)-E\left[\log|\frac{\partial g}{\partial X}|\right]\\ \Rightarrow H\left(X|y\right)-H\left(y|X\right)=-E\left[\log|\frac{\partial g}{\partial X}|\right] \end{array} \end{array}$$

Furthermore, *I*(*X, y*) attains its maximum (i.e., preservation of the information of the original random variable, X) when $\frac{\partial I\left(X,y\right)}{\partial g\left(X\right)}=0$ i.e., its slope with respect to the transformation *g* is zero. Moreover, *g*:*X*→*y* without any external effect (e.g., difference between the input and output signals due to the effect of the medium). Therefore, the amount of uncertainty in *y*, given *X* must not exceed that of *X*:

(9) $$\begin{array}{l} H\left(y|X\right)\leq H\left(X|y\right)\Rightarrow H\left(X|y\right)-H\left(y|X\right)\geq 0 \end{array}$$

Using Equations (8) and (9), we have:

(10) $$\begin{array}{l} -E\left[\log|\frac{\partial g}{\partial X}|\right]\geq 0\Rightarrow 0\leq |\frac{\partial g}{\partial X}|<1 \end{array}$$

and $\frac{\partial g}{\partial X}$ i.e., the slope is $\frac{1}{\sigma_{X}}$ or $\frac{1}{X_{max}-X_{min}}$, given the z-normalization or the scaling transformations, respectively. Therefore, Equation (10) holds iff 0 ≤ σ_*X*_ < 1, σ_*X*_ ≠ 0 and −1 ≤ *X*_*max*_ − *X*_*min*_ < 1, *X*_*max*_ − *X*_*min*_ ≠ 0. However, this requirement is unwarranted since *X*_*max*_ − *X*_*min*_ ∈ ℝ and σ_*X*_ ∈ ℝ.

       ■

Although averaging provides an effective tool for the analysis of certain aspects of the naturalistic stimuli (e.g., neural coupling between the speaker and the listener, Stephens et al., 2010), this feature extraction strategy is ill-suited for capturing the essential information pertinent to the responses to such stimuli in their broader sense (e.g., storytelling and conversation, Spiers and Maguire, 2007; Erceg-Hurn and Mirosevich, 2008; Hasson and Honey, 2012; Wilcox, 2012, 2017; Wehbe et al., 2014; Liu et al., 2017; Rousselet et al., 2017). In fact, Ben-Yakov et al. (2012) show the shortcoming of the averaging-based methods for detecting responses that are highly context dependent (e.g., listening to a story), resulting in worsening the effective signal-to-noise ratio. Moreover, Haynes and Rees (2007) claim that the averaging of the brain activity loses its functional state at any given point in time. We formalize these observations through following Proposition.

Proposition 2.3. *Averaging results in an information representation that tends to the overall expected value of the underlying distribution of a given time series*.

*Proof*. Let *X* represent a given time series. Furthermore, let *x*_*i*_ ⊆ *X* be the *i*th segment of *X* with length *k* i.e., ||*x*_*i*_|| = *k* ≤ ||*X*||, and $\bar{x_{i}}\to \mu_{X}$ as *k* → ||*X*||, i.e.,

(11) $$\begin{array}{l} \underset{k\to ||X||}{\lim}P\left(|\bar{x_{i}}-\mu_{X}|>\epsilon \right)=0 \end{array}$$

where $\bar{x_{i}}$ and μ_*X*_ are the sample mean of *x*_*i*_ and the mean of the time series *X. P*(.) gives the probability of the occurrence of its argument and |.| returns the absolute difference between its two parameters. It is apparent that there exists $\frac{\left||X|\right|}{k}$ such segments and:

(12) $$\begin{array}{l} \mu_{X}=\frac{1}{\frac{\left||X|\right|}{k}}\overset{\frac{\left||X|\right|}{k}}{\underset{i=1}{\sum }}\bar{x_{i}} \end{array}$$

due to the law of large numbers. Therefore, these values reveal the general trend of their respective segments, thereby tending to the overall trend i.e., the expected value of *X* as ||*X*|| → ∞:

(13) $$\begin{array}{l} \underset{||X||\to \infty }{\lim}P\left(\mu_{X}-\frac{1}{\frac{\left||X|\right|}{k}}\overset{\frac{\left||X|\right|}{k}}{\underset{i=1}{\sum }}\bar{x_{i}}>\epsilon \right)=0 \end{array}$$

       ■

A common practice in the literature pertinent to analysis of the effect of the stimuli on the hemodynamic and/or the neural activity of the human subjects is the application of the sliding window on the given time series that is associated with these signals. In such a setting, the two consecutive segments that are extracted from the time series *X* share an overlapping subsegment of length 0 ≤ *n* ≤ ||*X*||−1. Here, zero indicates that the consecutive segments do not overlap, whereas the upper limit means that the original series divided into two with only a single data point uncommon between these segments (i.e., the minimum requirement for the segmentation of ||*X*|| into two distinct segments). An immediate implication of Proposition 0.0.3 is that the larger the overlap between the two consecutive segments is, the closer their respective expected values are. In other words, the overlap between the two segments results in the greater loss of the variational information. Furthermore, it indicates that the averaging becomes less effective if the events that are associated with a stimulus are subtle in nature (e.g., emotional cues reflected in a story) as opposed to a rather trigger-based event (e.g., appearance of a specific digit on a blank screen at a well-defined, predetermined point in time).

On the other hand, Eden and Kramer (2010) note the Fano factor (Fano, 1947) as an important characterization of neural spiking. In addition, they point out that such action potentials (i.e., neuronal spikes) often exhibit a high variability. What the utilization of slope/mean of a NIRS time series falls short on representing is indeed this variability. It is apparent that these action potentials are in fact the underlying dynamics of the brain activity, thereby reflecting the changes in the local hemodynamic such as blood oxygen content and its subsequent level of consumption (Gusnard and Raichle, 2001). In other words, fluctuations that are observed in NIRS time series are in fact an indirect representation of the responses that are induced by these action potentials as their underlying dynamics of the brain activity. In the following Proposition, we demonstrate the ability of the linear estimate of differential entropy (DE) (Cover and Thomas, 2006; Stone, 2015) in explicitly capturing this variability.

**Theorem 2.4**. *The expected rate of change in the linear estimate of differential entropy of a time series X is inversely proportional to the Fano factor, F*.

*Proof*. Linear estimate of the differential entropy of a random variable *X* is (Cover and Thomas, 2006; Xiong et al., 2017):

(14) $$\begin{array}{l} H\left(X\right)=\frac{1}{2}\log_{b}\left(g\left(X\right)\right) \end{array}$$

where,

(15) $$\begin{array}{l} \begin{array}{c} g\left(X\right)=2\pi e\sigma_{X}^{2}\\ =2\pi e\frac{\underset{x\in X}{\sum }\left(x^{2}-2x\mu_{X}+\mu_{X}^{2}\right)}{N}\\ =2\pi e\left(\frac{\underset{x\in X}{\sum }x^{2}}{N}-\frac{2\mu_{X}\underset{x\in X}{\sum }x}{N}+\frac{N\mu_{X}^{2}}{N}\right)\\ =2\pi e\left(\frac{\underset{x\in X}{\sum }x^{2}}{N}-\mu_{X}^{2}\right) \end{array} \end{array}$$

with *N* = ||*X*|| is the cardinality of X. Using Equation (15), derivative of *H* with respect to μ_*X*_ is:

(16) $$\begin{array}{l} \;\frac{\partial H}{\partial \mu_{X}}=\frac{1}{2}\frac{\frac{\partial g}{\partial \mu_{X}}}{ln(b)g(X)}\\ \;=\frac{1}{2}\frac{\frac{\partial }{\partial \mu_{X}}[(2\pi e(\frac{\sum_{x\in X}x^{2}}{N}-\mu_{X}^{2})]}{ln(b)2\pi e\sigma_{X}^{2}}\\ \;=\frac{-4\pi e\mu_{X}}{ln(b)4\pi e\sigma_{X}^{2}}\\ =\frac{-\mu_{X}}{ln(b)\sigma_{X}^{2}}=-\frac{\mu_{X}}{\sigma_{X}^{2}}=-F^{-1},\;b=e \end{array}$$

       ■

### 2.2. Tasks

We conducted two series of experiments, referred to as *Working Memory Experiment* (WME) and *Conversational Task Experiment (CTE)*, hereafter. We chose WME due to its utility in analytical studies of the cognitive loads on the working memory of human subjects. Moreover, we considered a number of working memory tasks with well-established differences in their imposed cognitive load on the WM (Cui et al., 2011; Verner et al., 2013; Fishbum et al., 2014; Herff et al., 2014), thereby allowing for investigation of the ability of DE in capturing the underlying dynamics and variation of the brain activity in response to differing cognitive loads. On the other hand, we chose CTE to analyze the utility of the linear estimate of DE in response to more subtle variation in brain activity in response to naturalistic stimuli (e.g., conversation, listening to a story, etc.). Results of these analyses contribute in realization of the utility of linear estimate of DE in analytical as well as decoding domains of NIRS-based modeling of brain activity of human subjects. Description of WME and CTE are as follows.

1. WME: It consisted of four different working memory tasks, namely, Listening Span Test (LST) (Osaka et al., 2003), N-Back (B), Stroop (S), and Mental Arithmetic (M). Each of these working memory (WM) tasks consists of two subtasks. Their descriptions are as follows.
  - LST: There were two subtasks, namely, L1 and L2, consisting of two and three sentences, respectively. These sentences were readout to the participants sequentially. Participants were instructed to judge the validity of each sentence once its reading was over [e.g., Sun sets in the west. (yes/no?)]. Once reading of sentences of a given subtask were complete, participants were required to recall the first word of each of the sentences. This resulted in two and three words recall in case of L1 and L2, respectively.
  - B: It included a one-back (B1) and a two-back (B2) WM tasks. We used a recorded call-out of numerical sequences (0 through 9) in which participants were required to respond to sequential (i.e., B1) and every-other (i.e., B2) repetition of these digits via clicking the arrow keys on the computer keyboard.
  - S: It contained two subtasks, involving two-color (i.e., S1) and three-color (i.e., S2) streams. Both of these subtasks consisted of a sequence of twenty words (i.e., name of a color such as “red,” or “green”) that were randomly matched/mismatched with their corresponding colors (e.g., word “red” was shown with its matching color, red, or a mismatching color such as blue). We used the color/word “red, blue“ in S1 and “red, blue, green” in S2.
  - M: It comprised of two subtasks, requiring the mental addition of a two-digit number with a single-digit number (M1) and two two-digit numbers (M2), respectively. There were four addition operations in each of these subtasks, resulting in eight arithmetic operations in total. In case of M2, half of these operations resulted in carryover.
  Every subject participated in all of these four WM tasks. We acquired a 1-min-long resting data of the participants (with their eyes closed) prior to start of each subtask which was followed by its corresponding task. Furthermore, we randomized the ordering of these WM tasks while keeping the order of their corresponding subtasks intact for all participants. We used the PsychoPy (Peirce, 2003) in WME.
2. CTE: This paradigm comprised of 3-min-long conversation sessions in which we discussed four different topics (two easy and two difficult) with the participants (in Japanese). We communicated with our participants through minimalist anthropomorphic android, the Telenoid, to eliminate the potential effect of human characteristics such as gender and age. This resulted in four separate sessions, per participant. In every session, we began with acquiring a 1-min-long resting data, followed by its corresponding 3-min-long experimental session. We kept the content of conversations intact in all sessions. Every subject participated in all of these settings. However, we randomized the order of the easy/difficult among our participants. We provided our participants with a 1-min-long resting break (while staying at their seat with their eyes closed) prior to the commencement of each of these session. We maintained an approximately 1.2 m distance between the seat of the participant and the Telenoid. A male person conversed with our participants in all four conversational sessions.

### 2.3. Subjects

1. WME: Thirteen young adults (nine females and four males, *M* = 21.87, SD = 2.61) participated in these tasks.
2. CTE: Our participants included twenty two individuals (fourteen females and eight males, *M* = 46.55, SD = 10.62).

All participants were right-handed [confirmed using FLANDERS (Nicholls et al., 2013) handedness questionnaire], were free of neurological and psychiatric disorders, and had no history of hearing impairment. Prior to the data collection, we received approval (approval code: 16-601-1) from the ethical committee at the Advanced Telecommunications Research Institute International (ATR), Kyoto, Japan. All subjects gave written informed consent in accordance with the Declaration of Helsinki. Subjects were seated on an easy armchair in the sound-attenuated experimental room, with instructions to fully relax and their eyes closed while resting.

### 2.4. Data acquisition

**Figure 1:**
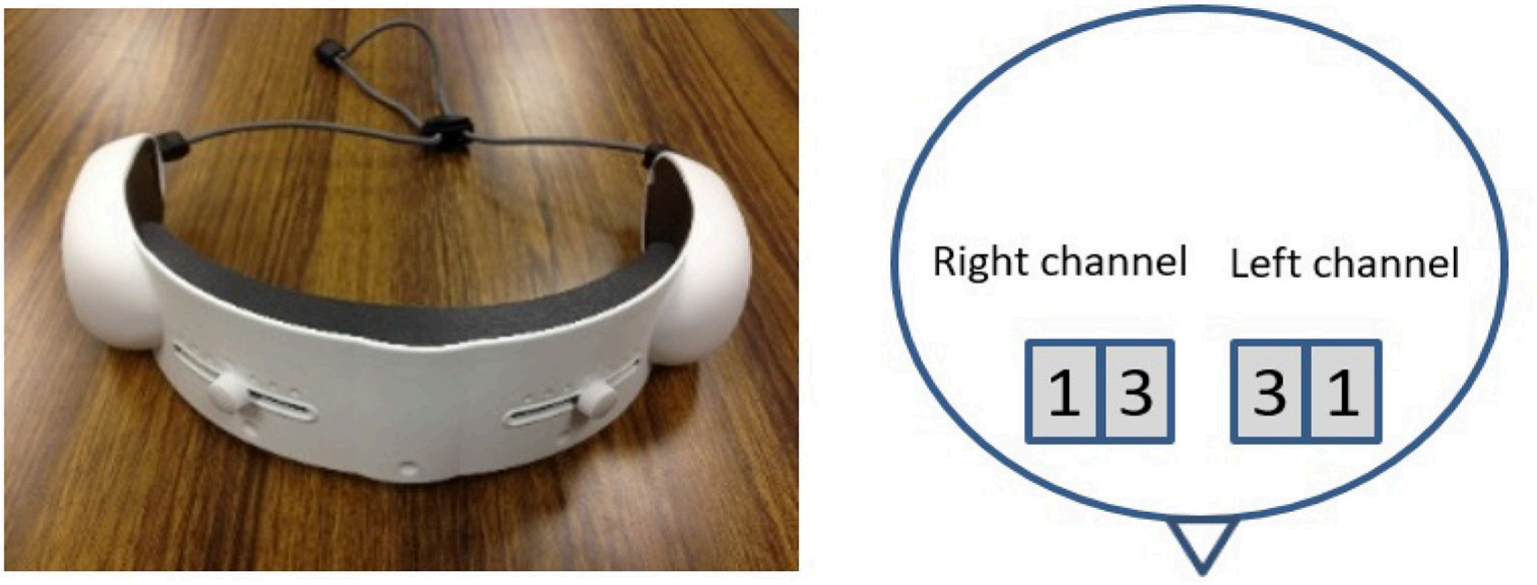
The NIRS device **(Left)** along with the schematic of the locations of the left and right channels associated with the data collection procedure during the experiment **(Right)**. The numbered squares refer to the left and right channels, respectively.

We used Near-Infrared Spectroscopy (Ferrari and Quaresima, 2012; Dix et al., 2013) to collect the brain activities in the frontal area of our participants. We acquired the NIRS time series of our participants using a wearable optical topography system “HOT-1000,” developed by Hitachi High-Technologies Corporation (please refer to Figure 1). Participants wore this device on their forehead in our experiments. This device collects data through four channels (i.e., *Left*_1_, *Left*_3_, *Right*_1_, and *Right*_3_, as shown in Figure 1). Furthermore, it allows for recording of the measurement of brain activity through detection of total blood flow via emitting a wavelength laser light (810 nm) at 10 Hz sampling frequency. The postfix numerical values that are assigned to these channels specify their respective source-detector distances. In other words, *Left*_1_ and *Right*_1_ have a 1.0 cm and *Left*_3_ and *Right*_3_ have 3.0 cm source-detector distances, respectively. Findings in the literature on brain region activation during memory and language processing suggest a left-lateralized activation in both genders with higher specificity in females (Haut and Barch, 2006; Li et al., 2010). Therefore, we primarily utilize the NIRS time series that is acquired through the channel *Left*_3_ of this device in the present study. It is worth noting that the source-detector distance of 3.0 cm is adequate for proper data acquisition of cortical brain activity using NIRS-based devices (Ferrari and Quaresima, 2012; León-Carrión and León-Domínguez, 2012; Dix et al., 2013).

### 2.5. Data preprocessing

First, we normalized the data corresponding to the selected NIRS channel via subtracting the mean of the 1 min resting period as a baseline from its data. Next, we applied a one-degree polynomial butter worth filter on this normalized data with 0.01 Hz and 0.6 Hz for low and high bandpass values, respectively. This was followed by its linear detrending.

### 2.6. Analyses

After data preprocessing step, we applied Wilcoxon signed-rank test on calculated features of time series data of our participants in both, WME and CTE experimental paradigms, to investigate the utility of different feature spaces in capturing potential differences in NIRS time series of brain activity. It is worth noting that we chose this non-parametric test to avoid any assumption on the underlying distribution of the data, as it is the case for two-sample *t*-test for example.

**Figure 2:**
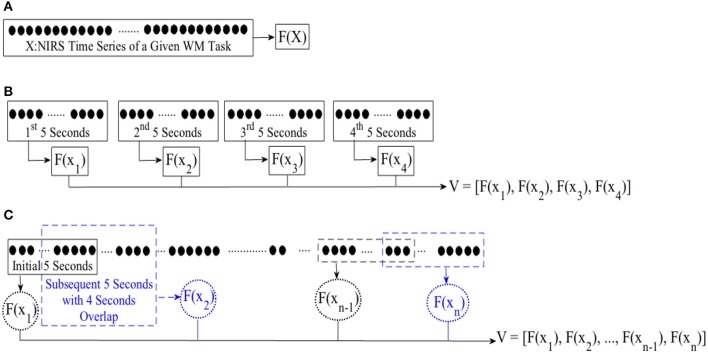
Feature extraction from **(A)** entire NIRS time series **(B)** consecutive non-overlapping windows of length 5-s **(C)** first initial window of 5-s and following with subsequent 1-s moving windows (i.e., 4 s of overlap between consecutive sequences). *F*(.) computes a desired feature from its argument, namely, differential entropy (DE), mean, slope, combination of mean & slope, or moving average. *X* is the entire time series of brain activity. *x*_*i*_ ∈ *X* refers to *i*th 5-s-long segment of a given time series. *V* represents the feature vector, resulting from application of an adapted feature extraction strategy. For instance, *F*(*x*_1_) implies calculating mean of the 1st 5-s-long segment while adapting mean feature extraction strategy with *V* as its resulting mean-based feature vector. We used mean value of elements of *V* for statistical analyeses (i.e., both WME and CTE). On the other hand, we use *V* in its vector form during cluster analysis of brain activity pertinent to CTE.

In case of WME, we first computed the mean, combined mean & slope, moving average, and DE features from the entire time series of corresponding subtasks, per participant. Figure 2A, illustrates this process. For combined mean & slope computation, we computed the mean and the slope of the entire time series, separately, and combined them into a single vector. We considered the slope of the fitted line to a given time series sequence for the latter. For moving average computation, we started with the first 5-s-long segment of a given NIRS time series and applied a 1-s moving window (i.e., 4 s of overlap between consecutive segments), calculating the mean of each of these segments. Figure 2C, shows this process. We used the mean of these averages (i.e., mean value of entries of *V* in Figure 2C) in our analysis. Furthermore, we used the normalized values of these features in comparative analysis of effectiveness of different feature spaces after application of normalization.

In case of CTE, we segmented each time series data into non-overlapping 5-s-long segments. For each segment, we extracted a feature using mean, mean & slope, moving average, and DE. Figure 2B, depicts this process. For combined mean & slope computation, we computed the mean and the slope (i.e., per segment), separately, and combined them into a single vector. We considered the slope of the fitted line to a given 5-s-long time series segment for the latter. Then, we computed the averaged mean & slope vector of these 5-s-long segments. We followed the same protocol as in WME for computing moving average (i.e., Figure 2C). We applied Wilcoxon signed-rank test on mean value (i.e., mean of entires of *V* in Figure 2B in case of mean, mean & slope, and DE and Figure 2C in case of moving average) of these extracted features.

**Figure 3:**
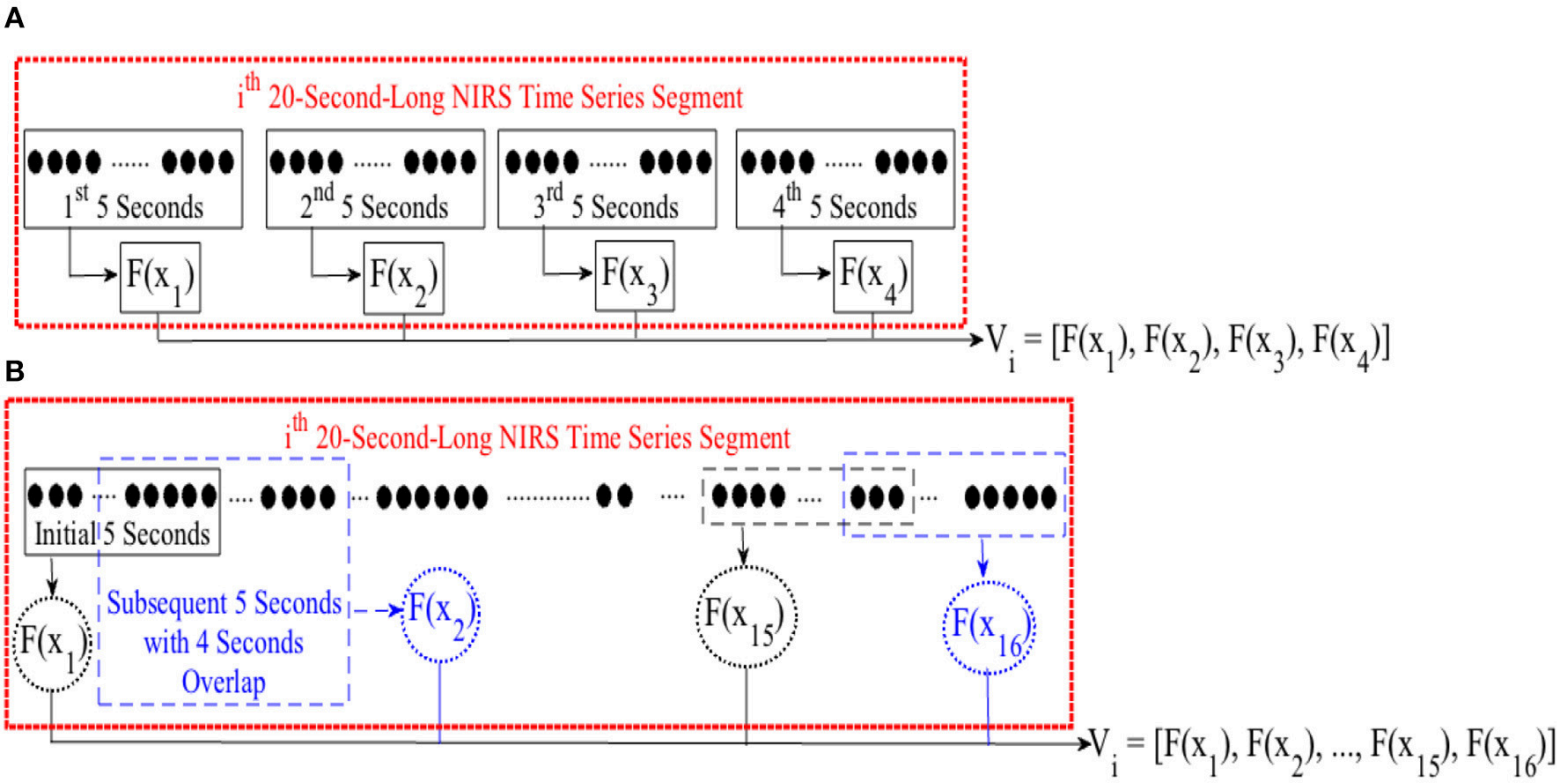
**(A)** Feature calculation for every 5-s-long non-overlapping sequence of *i*th 20-s-long segment of NIRS time series of brain activity. This results in a feature vector *V* of length four in case of mean or DE feature spaces and length eight in case of combined mean & slope feature extraction strategy. **(B)** Extracting moving average feature vector. We started with first 5-s-long segment of NIRS time series data and applied a 1-s-long moving window (i.e., 4 s of overlap between consecutive sequences). For every 5-s-long segment, we computed the mean value. This resulted in a feature vector *V* of length sixteen. *F*(.) computes a desired feature, namely, differential entropy (DE), mean, slope, combination of mean & slope, or moving average from its argument, *X. x*_*j*_ ∈ *X*_*i*_ refers to *j*th 5-s-long segment of *i*th 20-s-long NIRS segment. *V*_*i*_ represents the feature vector of this *ith* 20-s-long segment. We applied K-mean (Liao, 2005) clustering (with two centroids) on these feature vectors during cluster analysis of CTE.

For cluster analysis in **Figure 5**, we segmented each time series data pertinent to conversational task (i.e., CTE) into 20-s-long non-overlapping segments. For each segment, we computed a feature for every 5-s-long non-overlapping data sequence, resulting in a feature vector of length four in case of mean and DE (i.e., eight in combined mean & slope) for each 20-s worth of NIRS time series. Figure 3A, illustrates this process for the *i*th 20-s-long segment. On the other hand, we started with the first 5-s-long subsegment of each of these 20-s-long segments and applied 1-s moving window (i.e., 4 s of overlap between every consecutive subsegments) to calculate the feature vector based on moving average strategy. This resulted in a feature vector of length sixteen, per 20-s-long worth of data (Figure 3B). Next, we applied K-mean (Liao, 2005) algorithm (with two clusters) on these feature vectors (i.e., *V* in Figure 2A through Figure 2C).

## 3. Results

In this section, We validate our mathematical proofs on effectiveness of the linear estimate of differential entropy (DE) of NIRS time series in contrast with the conventional averaging-based feature extraction strategies through statistical analysis (Wilcoxon test) of the brain activity of human subjects. Our analyses pertain to two different experiments, namely, Working Memory Tasks Experiment (WME) and Conversational Tasks Experiment (CTE). Results in each of these sections are structured as follows.

1. WME: section 3.1.1 includes the analyses of Listening Span Test (LST), N-Back, Stroop, and Mental Arithmetic (MA) Working Memory (WM) tasks where each of these tasks comprised of two subtasks of different cognitive loads. In this subsection, we use features that are calculated based on actual value of NIRS time series data of the participants. On the other hand, we present results of this test on normalized NIRS time series data of the participants in section 3.1.2.
2. CTE: section 3.2 provide evidence on ability of linear estimate of DE in resolving the shortcoming of the averaging-based features in case of naturalistic stimuli (Spiers and Maguire, 2007; Erceg-Hurn and Mirosevich, 2008; Ben-Yakov et al., 2012; Hasson and Honey, 2012; Wilcox, 2012; Wehbe et al., 2014; Liu et al., 2017; Rousselet et al., 2017) via analyses of the brain activity of human subjects involved in four conversations with the content of whose varied in their level of difficulty.

### 3.1. WME

#### 3.1.1. Original feature spaces

Wilcoxon test on application of mean as adapted feature space implied significant difference between two subtasks of Listening Span Test (LST) Working Memory (WM) tasks, i.e., L1 and L2 [*p* < 0.001, *T*_(24)_ = −4.31], as well as subtasks S1 and S2 in Stroop WM [*p* < 0.001, *T*_(24)_ = −6.65]. However, it indicated non-significant with respect to Mental Arithmetic M1 and M2 [*p* = 0.23, *T*_(24)_ = 1.20] as as well as N-Back B1 and B2 [*p* = 0.47, *T*_(24)_ = 0.72] WM tasks.

Similarly, combination of mean & slope indicated significant difference between L1 and L2 [*p* < 0.001, *T*_(24)_ = −4.31], as well as S1 and S2 [*p* < 0.001, *T*_(24)_ = −6.65]. However, it implied non-significant with respect to M1 and M2 (*p* = 0.231124) as well as B1 and B2 [*p* = 0.47, *T*_(24)_ = 0.72].

Although, application of moving average showed significant difference between L1 and L2 [*p* < 0.001, *T*_(24)_ = −4.31] as well as S1 and S2 [*p* < 0.001, *T*_(24)_ = −6.65], it implied non-significant with regards to M1 and M2 [*p* = 0.24, *T*_(24)_ = 1.17] as well as B1 and B2 [*p* = 0.81, *T*_(24)_ = −0.23].

On the other hand, DE indicated significant differences between L1 and L2 [*p* < 0.001, *T*_(24)_ = −4.31], S1 and S2 [*p* < 0.01, *T*_(24)_ = −4.72], as well as M1 and M2 [*p* = 0.03, *T*_(24)_ = −2.55] while suggesting non-significant difference between B1 and B2 [*p* = 0.64, *T*_(24)_ = −0.47]. Table 1 provides summary statistics of these results.

**Table 1:** Working Memory Experiment (WME): Mean (M), Standard Deviation (SD), and Standard Error (SE) of Moving Average, DE, Mean, and combined Mean & Slope feature spaces with respect to N-Back (B1 and B2), Listening Span Test (L1 and L2), Stroop (S1 and S2), and Mental Arithmetic (M1 and M2) WM tasks.

**WME**	**Moving Average**			**DE**			**Mean**			**Mean & Slope**		
	***M***	**SD**	***SE***	***M***	**SD**	***SE***	***M***	**SD**	***SE***	***M***	**SD**	***SE***
B1	763.20	2.54	0.48	10.39	0.20	0.04	374.54	0.13	0.02	374.54	0.13	0.02
B2	762.81	3.71	0.70	10.39	0.17	0.03	376.85	48.57	0.09	374.52	0.09	0.02
M1	87.83	26.95	5.01	5.86	1.12	0.21	42.60	13.27	2.46	42.62	13.26	2.46
M2	80.44	29.06	5.40	6.68	1.03	0.19	39.10	14.25	2.65	39.12	14.25	2.64
S1	106.03	14.70	2.68	6.22	0.77	0.14	50.90	7.23	1.32	50.91	7.23	1.32
S2	212.98	27.80	5.08	7.51	1.24	0.23	105.82	14.12	2.58	105.82	14.12	2.58
L1	286.48	33.48	9.29	7.61	1.95	0.54	142.85	16.61	4.61	142.85	16.61	4.61
L2	771.07	0.69	27.93	10.47	0.31	0.09	376.85	48.57	13.47	376.85	48.57	13.47

*These descriptive statistics are based on actual NIRS time series data of the participants (i.e., after preprocesing of the NIRS data and prior to application of normalization)*.

#### 3.1.2. Normalized feature spaces

**Figure 4:**
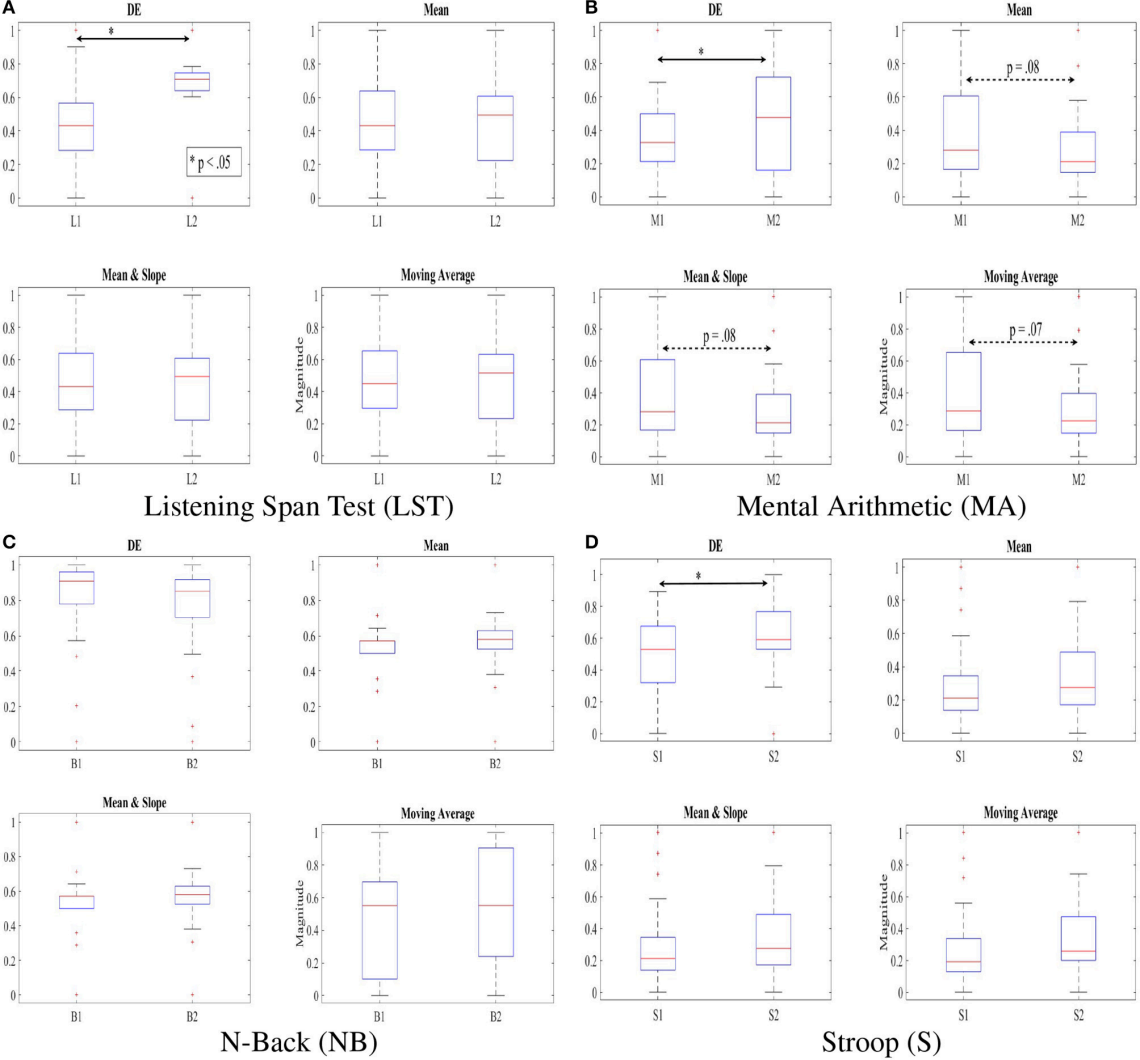
DE, mean, combined mean & slope, and moving average of the feature vectors of NIRS time series of our participants, scaled to fit within [0.…1] interval using $\frac{v-min\left(V\right)}{max\left(V\right)-min\left(V\right)},\;\forall v\in V$. Subtasks with significant differences are marked by asterisk. The dotted double-arrow lines indicate the tendency between the subtasks, as implied by a given feature space. Non-significant effect of inclusion of slope is apparent, comparing “Mean” and “Mean & Slope” entries in this table. **(A)** Listening Span Test (LST). **(B)** Mental Arithmetic (MA). **(C)** N-Back (NB). **(D)** Stroop (S).

Figure 4 shows discrimination of two subtasks of each of these WM tasks based on application of DE, mean, combined mean & slope, as well as moving average as feature extraction strategies on normalized NIRS time series data of the participants. In particular, Figures 4A,B show the ability of DE in capturing the significant different between the two subtasks in LST, i.e., L1 and L2 [*p* < 0.05, *T*_(24)_ = −2.41, SD = 0.26] and Stroop, i.e., S1 and S2 [*p* < 0.05, *T*_(24)_ = −1.99, SD = 0.22] where mean [LST: *p* = 0.96, *T*_(24)_ = 0.05, SD = 0.27, Stroop: *p* = 0.15, *T*_(24)_ = −1.42, SD = 0.24], mean & slope [LST: *p* = 0.15, *T*_(24)_ = −1.43, SD = 0.27, Stroop: *p* = 0.15, *T*_(24)_ = −1.43, SD = 0.24], as well as moving average [LST: *p* = 1.0, *T*_(24)_ = 0.0, SD = 0.28, Stroop: *p* = 0.08, *T*_(24)_ = −1.65, SD = 0.23] were unable to determine such differences. Most interesting is the result of utilization of these features in analysis of mental arithmetic (MA) where DE indicated an apparent significant between M1 and M2 (*p* < 0.05, *T*_(24)_ = −2.02, SD = 0.28] while other features implied a tendency [Mean: *p* = 0.15, *T*_(24)_ = 1.43, SD = 0.28, Mean & Slope: *p* = 0.15, *T*_(24)_ = 1.43, SD = 0.28, Moving Average: *p* = 0.14, *T*_(24)_ = 1.48, SD = 0.28].

### 3.2. CTE

Wilcoxon test implied non-significant between NIRS time series of the participant with respect to the topic of conversation, i.e., easy and hard, using mean [*p* = 0.37, *T*_(746)_ = 0.76], moving average [*p* = 0.41, *T*_(746)_ = 0.83], as well as combined mean & slope [*p* = 0.32, *T*_(746)_ = 0.69]. However, it indicated significant based on application of DE on these time series [*p* < 0.001, *T*_(746)_ = 4.00]. Table 2 provides the summary statistics of the mean- and DE-based features in conversational task experiment.

**Table 2:** Conversational Tasks Experiment (CTE): Mean (M), Standard Deviation (SD), and Standard Error (SE) of Moving Average, DE, Mean, and combined Mean & Slope feature spaces with respect to easy and hard conversational topics during CTE.

**CTE**	**Moving Average**			***DE***			**Mean**			**Mean & Slope**		
	***M***	**SD**	***SE***	***M***	**SD**	***SE***	***M***	**SD**	***SE***	***M***	**SD**	***SE***
Easy	−20.31	179.05	9.46	1.73	0.84	0.04	−14.87	63.25	5.16	−14.83	61.20	7.16
Hard	−15.82	149.23	7.56	2.73	1.99	0.07	−8.62	76.61	9.26	−8.90	78.67	10.26

*These descriptive statistics are based on actual NIRS time series data of the participants*.

## 4. Discussion

In this article, we argued that averaging-based feature extraction strategies that are inspired by high degree of linearity in NIRS time series of brain activity of human subjects results in suboptimal solution in capturing the variational information of these signals, thereby limiting the reliability of an adequate conclusion on their outcomes. Alternatively, we proposed the linear estimate of differential entropy of these time series as a natural representation of such information. We provided evidence for our claim through theoretical and empirical comparative analyses of the application of these features on NIRS data pertinent to a number of WM tasks with varying level of cognitive loads. Concretely, we demonstrated the utility of DE in contrast with mean, combination of mean & slope, and moving average feature spaces in analysis as well as differentiation of subtasks of a several WM tasks into their corresponding classes. These WM tasks included Listening Span Tests (L1 and L2), Stroop (S1 and S2), N-Back (B1 and B2), and Mental Arithmetic (M1 and M2). We further showed the confounding effect of these averaging-based feature extraction strategies via analysis of a naturalistic conversational tasks with differing level of difficulty in their respective topics, thereby providing evidence on inability of these features in representing the significance in responses of our participants to varying contextual complexity of conversational topics. Subsequently, we presented the sensitivity of DE in extracting this information. Moreover, we illustrated the substantial similarities between distribution of data based on mean and combination of mean & slope, thereby indicating the negligible contribution of the slope in representation of the information content of the brain activity of human subjects in response to WM as well as naturalistic conversational tasks with varying degree of difficulty in their topics.

Although we found similar indication of non-significant difference between N-back subtasks through application of DE as well as averaging-based feature spaces of the NIRS time series of the brain activity of the participants, we suggest that such a similarity is due to the significant resembling dynamics of these subtasks, and consequently, their equivalence in imposed cognitive loads on human subjects. This claim is due to comprehensive results in study and analysis of N-Back WM task (Owen et al., 2005; Fishbum et al., 2014). This observation is further supported by non-significant difference in induced level of complexity by B1 and B2 WM tasks on brain activity of our participants, as implied through result of multiscale entropy (MSE) analysis (Costa et al., 2002, 2005) of NIRS time series of their brain activity (please refer to Appendix for further details).

Fano factor (Fano, 1947) characterizes the neural spiking as a deviation of activation of neural population from their expected spiking rate. In other words, it signifies the expected brain activity in response to a given stimulus as per variation that is exhibited by neural population. In this regards, application of averaging-based feature spaces is equivalent to constraining such an activity within one standard deviation of its expected or average value (i.e., σ^2^ ≤ 1 in $F=\frac{\sigma^{2}}{\mu }$, Fano, 1947). It is apparent that such a constraint is unwarranted, resulting in an information loss on functional state of neural population (Haynes and Rees, 2007). This interpretation finds evidence in recent findings that imply the effect of working memory tasks is significantly captured by the variance of the information content of neural activity (Lundqvist et al., 2018). In fact, change in brain activity of human subjects during WM tasks is quantified by its variational information (Miller, 2001) than its average activity or expectation. On the other hand, the mathematical bound between linear estimate of DE and Fano factor, as shown through Theorem 0.0.4, along with our empirical results on NIRS time series of brain activity during WM and conversational tasks suggest the effectiveness of DE in quantification of this variational information of brain activity of human subjects. However, further validation of these results on a larger sample size for drawing an informed conclusion on utility of DE in analysis of NIRS time series of brain activity of human subjects during WM tasks is necessary.

Another source of evidence on significance of the variational information of brain activity is due to the results of the analyses of physiological systems from perspective of their dynamical complexity. Research suggests that increase in complexity is an inherent attribute of healthy physiological systems (Lipsitz and Goldberger, 1992; Costa et al., 2002, 2005; Goldberger et al., 2002a,b; Takahashi et al., 2009). Additionally, it implies that such an increase in complexity strongly correlates with such cognitive functions as attention, memory, mental manipulation, verbal fluency, and language (Manor and Lipsitz, 2013; Yang et al., 2013). Interestingly, an increase in complexity implies a direct correspondence to variational information that is exhibited by such dynamical and physiological systems (Zhang, 1991; Fogedby, 1992; Gao et al., 2015). On the other hand, feature spaces such as event-specific mean activity and slope (i.e., a regression coefficient) follow the key assumption of linear model that the neuronal dynamics and their transients can be ignored (Friston et al., 1994a). As a result, they are suitable for scenarios in which neural responses are monotonic and, subsequently, fail if monotonicity in variational information is violated (Pouget et al., 2016).

Research on working memory (WM) (Baddeley, 2003, 2012) suggests activation of prefrontal cortex (PFC) in a variety of tasks, ranging from mental tasks with high cognitive loads (Cohen et al., 1997; Tsujimoto et al., 2004; Owen et al., 2005) (i.e., change in activation in comparison with general/baseline activity, Gusnard and Raichle, 2001) to change in mental state (Ozawa et al., 2014; Sato et al., 2014) and social cognition such as emotional responses (Wolf et al., 2010) and story comprehension (Mar, 2011). An important implication of these results is the activation of some similar regions in PFC in response to these tasks. They are dorsolateral (MacPherson et al., 2002), ventrolateral, and medial PFC (Owen et al., 2005; Mar, 2011; Fishbum et al., 2014; Ozawa et al., 2014). Given the increased activity of PFC by mental tasks (Cohen et al., 1997; Tsujimoto et al., 2004; Owen et al., 2005; Ozawa et al., 2014; Sato et al., 2014), social cognition (Wolf et al., 2010) and story comprehension (Mar, 2011) along with these similar PFC activated regions in response to such tasks, it is plausible to attribute the ability of DE in preservation of variational information of frontal brain activity in both, WM and conversational tasks, due to these common underlying activated regions (Owen et al., 2005; Mar, 2011; Fishbum et al., 2014; Ozawa et al., 2014). Considering the fact that DE is the average information content in a continuous time series data (Cover and Thomas, 2006; Avery, 2012; Stone, 2015), it is apparent that detected variation in such an information content summarizes the change of activity in these areas in response to stimuli (i.e., difficulty of mental tasks and topics of conversation in our case). Although configuration of recording channels of our NIRS device (please refer to Figure 1) to capture activity pertinent to PFC suggests further evidence in support of this observation, we are unable to confirm this presumption due to limited spatial resolution of our NIRS device. Therefore, future research to determine the credibility of this observation is required. It is also crucial to note that the ability of DE in preservation of variational information of tasks associated with WM does not warranty its utility as a universal NIRS feature, thereby necessitating further investigation to acquire insights on its performance with respect to NIRS time series of other mental states such as sleep and awake, relaxation and resting, and vigilance, to name a few.

**Figure 5:**
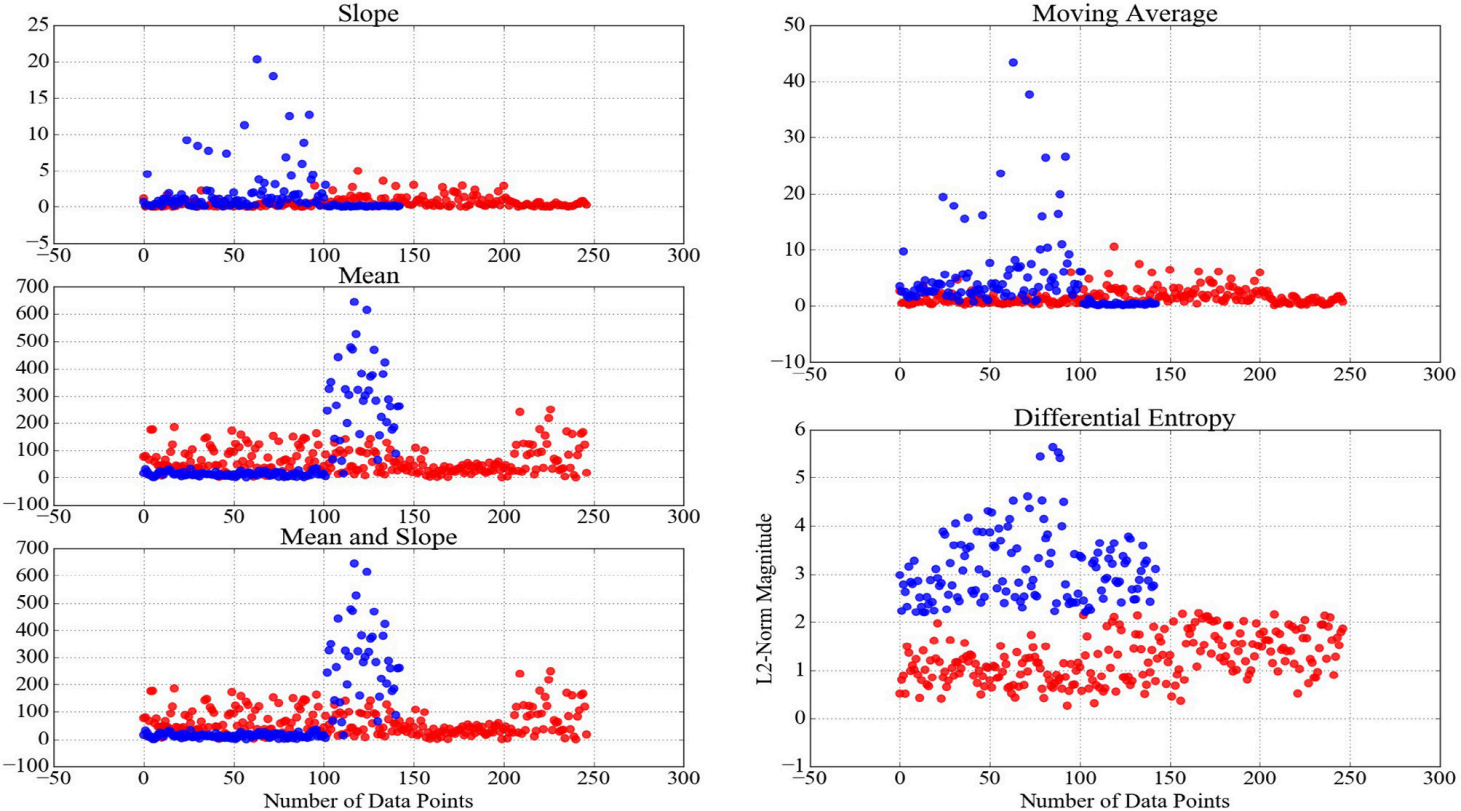
Clusters generated through application of K-mean clustering algorithm (Liao, 2005) using NIRS time series of brain activity of the participants during CTE. The red- and blue-colored data points in these clusters reflect their formation, given the magnitude (L2-norm in this case) of their corresponding feature vectors of 20-s-long NIRS time series during these conversations. The well-defined boundary between these clusters based on DE, as opposed to results of other feature extraction strategies, is apparent in this figure.

An important implication of analytical studies of pattern of brain activity of human subjects during cognitive tasks is their integration in real-life applications (Mitchell et al., 2008; Baucom et al., 2012; Naseer and Hong, 2015; Shin et al., 2016; Horikawa and Kamitani, 2017). It is apparent that feature extraction strategies play a pivotal role in effectiveness of such decoding applications. This effect becomes more crucial, considering the requirement of the transformation of these time series (e.g., baseline- and z-transformation, scaling the feature vectors within [0, …, 1] interval, etc.) prior to their decoding, thereby preventing the detrimental effect of bias and variance. Therefore, it is desirable for an adapted feature extraction strategy to be able to maximize the preservation of the variational information of these time series data after the application of such transformations. For instance, Xiong et al. (2017) emphasize the substantial effect of normalization on detecting a fine-grained alterations of the dynamical transitions of physiological systems across time and multiple scales. In this respect, Proposition 0.0.2 implied that the adaptation of the slope of the NIRS time series of brain activity results in loss of such variational information. On the other hand, Keshmiri et al. (2017) suggest the ability of DE of NIRS time series in outperforming the averaging-based feature spaces for decoding of the brain activity during N-Back working memory task. Whereas their results provide evidence on utility of DE in supervised learning paradigms, it is of significant importance to examine its ability in preservation of the information content of the brain activity in tasks with the underlying cognitive load of whose exhibit higher degree of non-triviality due to higher subjectivity of responses of human subjects (e.g., naturalistic scenarios such as conversation and story comprehension). Figure 5 shows two clusters that are generated using K-Mean clustering algorithm (Liao, 2005) on the feature vectors of the consecutive 20-s-long segments of NIRS time series of the brain activity of the participants during CTE. We used mean, slope, combination of mean and slope, moving average, and DE to generate these feature vectors to determine the utility of different feature extraction strategies in clustering data pertinent to naturalistic stimuli (i.e., difficulty of conversational topics in this case). The y-axis of these subplots represent the magnitude of the L2-norm of vectors of each of these feature spaces. It is worthy of note that cardinality of these clusters do not reflect a one-to-one correspondence between the number of easy and difficult conversational topics in CTE, as defined by the overall presupposition of these topics in our experimental paradigm. However, we expect to observe such an uneven grouping behavior of the L2-norms of the time series of the brain activity of the participants, given the substantial differences of the mental responses of the individuals to subtleties and emotional cues in naturalistic scenarios (Haynes and Rees, 2007; Spiers and Maguire, 2007; Wolf et al., 2010; Ben-Yakov et al., 2012; Hasson and Honey, 2012; Wehbe et al., 2014; Liu et al., 2017). Considering the changes in the magnitude of these feature vectors, as summarized by their respective L2-norms, as well as the result of our analysis on the significance of the topics of conversation, it is plausible to interpret the grouping phenomenon that is exhibited by these clusters as evidence in effectiveness of DE in modeling the variational information of the brain responses of the participants during these naturalistic cognitive tasks. This interpretation is strengthened via comparison of DE-based clusters in contrast with clusters that are formed by mean, slope, combination of mean & slope, and moving average. In this figure, negligible informational contribution of the slope of these time series (as it was observed in case of WME) is evident. It is worthy of note that although our preliminary result on performance of DE in clustering the brain activity of human subjects during conversational tasks suggest its potential for fine-grained summarization of the information content of such time series data, future research is crucial to further investigate its property in related WM tasks, thereby determining its utility through more rigorous experimental paradigms with higher objective validation strategies (e.g., comparison of its classification results in contrast with self-assessed responses of the participants in response to their undergone cognitive load, their degree of interest in the topic and/or their perceived level of difficulty of the task, etc.).

Apart from the capability of DE in capturing the variational information that is implicit in the responses of the brain activity of human subjects to mental tasks with varying cognitive loads, DE presents a reliable tool for quantitative measurement of the amount of information in these activities. Concretely, we found that the difference in the amount of information (measured in bit i.e., base 2 logarithm) in B1 (Mean = 5.10, SD = 1.08) was significantly above one standard deviation from L1 (Mean = 3.77, *SD* = 0.34), M1 (Mean = 2.59, SD = 1.19), and S1 (Mean = 2.68, SD = 1.46), as observed in their z-score differences (0.20, 1.33, −0.8, −0.73). Similarly, M2 (Mean = 2.69, SD = 1.18) had an amount of information that was significantly one standard deviation below L2 (Mean = 5.15, SD = 1.07), B2 (Mean = 5.11, SD = 1.07), and S2 (Mean = 3.67, SD = 0.62), where their z-scores were 0.83, 0.90, −1.23, and −0.41, respectively. Furthermore, we found that such a difference was non-significant in case of conversational tasks (Seniors: Mean = 1.75, SD = 0.84, Min = 0.04, Max = 4.27 and Juniors: Mean = 2.48, SD: 1.80, Min: 0.12, Max: 7.58), with their z-scores within one standard deviation. These values of the information content of the time series data of our participants that are in well-agreement with the theoretical boundaries that are proposed for the capacity of the information processing of human subjects (Miller, 2001) provide further insight on dynamics of these WM tasks and their consequential effect on brain activity of human subjects. Additionally, it is plausible to interpret these differences in amount of information as a measure of their respective cognitive loads on the brain activity of the human subjects during these mental tasks.

Proposal of the linear estimate of differential entropy as a feature extraction strategy for NIRS time series implies two assumptions. Firstly, it assumes a linearity of the underlying dynamics of the time series data under investigation (Kaiser and Schreiber, 2002; Lizier, 2014). This is consistent with the widespread use of linear models on the basis of the linear contributions from different sources (Mitchell et al., 2008; Huppert et al., 2009; Friston et al., 2011). Secondly, it assumes the normality of the underlying distribution of such data. Although the validity of this assumption is unwarranted, a number of results on modeling fMRI time series help interpret its utility, given the observed correlation between NIRS hemodynamic blood oxy/deoxy-genation hemoglobin and BOLD (Strangman et al., 2002; Okamoto et al., 2004; Toronov et al., 2007; Cui et al., 2011). In particular, Friston et al. (1994b) suggest that Poisson distribution well models the shape of the hemodynamic responses. Additionally, Boynton et al. (1996) show that gamma function is an effective tool in modeling hemodynamic responses in visual cortex. Furthermore, Aguirre et al. (1998) show that gamma function exhibits higher sensitivity in capturing the variability of these responses in an event-related sensorimotor task. The close relationship between the gamma and Poisson distribution is well understood. Concretely, for a random variable X ~ Γ(α, β), where α is an integer, we have $P\left(X\leq x\right)=P\left(Y\geq \alpha \right),\;Y\sim Poisson\left(\frac{x}{\beta }\right)$. Furthermore, if *Y*~*Poisson*(λ), then time until its *k* arrivals is the gamma function $\Gamma \left(k,\frac{1}{\lambda }\right)$, where *k* is the length of time until arrival of an event. Additionally, the correspondence between the Poisson and normal distribution is well realized. Precisely, an approximation of a Poisson distribution with parameter μ is the normal distribution N(μ,μ) (Rosner, 2016). In addition, the square root of a Poisson distributed random variable is approximately normally distributed (Johnson, 1993; Aguirre et al., 1998). Moreover, Poisson distribution forms a limiting case for a binomial distribution with a large number of observations and small probability of occurrence per observation while the z-score normalized observations of the latter correspond to the family of standard normal distribution. Furthermore, a binomially distributed random variable with parameters *n* and *p* is approximated by N(np,np(1-p)). These observations imply the potential of the presence of an underlying normality of the hemodynamic responses in blood oxy/deoxy-genation of the NIRS time series, thereby presenting the potential of the linear estimate of differential entropy of these time series to address the shortcoming of the averaging-based strategies in WM and naturalsitic tasks (Spiers and Maguire, 2007; Ben-Yakov et al., 2012; Hasson and Honey, 2012; Wehbe et al., 2014; Liu et al., 2017).

## 5. Conclusion

In this article, we studied the shortcoming of averaging-based feature extraction strategies in capturing the information content of brain activity of human subjects, as represented by NIRS time series, during WM and conversational tasks. Furthermore, we demonstrated the efficiency of linear estimate of differential entropy (DE) in quantification of information content of such time series, thereby presenting its correspondence with the underlying spiking neural activity.

We validated our mathematical analyses through application of these features in analysis of a number of working memory (WM) tasks. Our results suggested that DE shows higher sensitivity to brain activity of human subjects in comparison with mean, slope, combination of mean & slope, as well as moving average feature spaces. In addition, we showed that DE has higher sensitivity with regards to information gain through comparative analysis of its results in contrast with averaging-based feature spaces after the application of normalization and scaling. It is worth noting that such steps as baseline correction, normalization, and scaling are of crucial importance since they help refine data, thereby reducing detrimental effect of undesirable variation such as effect of outliers and biasing.

We validated the sensitivity of DE in capturing the variational information of time series of brain activity of human subjects to more naturalistic scenarios through comparison of its results with respect to averaging-based feature spaces on data pertinent to conversational time series. Whereas mean, slope, combination of mean & slope, as well as moving average feature extraction strategies implied non-significant in brain activity of human subjects in response to topics of conversation (i.e., easy topic such as daily activities vs. difficult such as conversation on a controversial topic), DE indicated significant difference in time series of brain activity in response to these conversational topics.

Although our results suggested the utility of DE in analysis of brain activity of human subjects pertinent to WM tasks with varying degree of cognitive loads, they do not imply its utility as a universal NIRS feature. For instance, it is necessary to examine its utility on NIRS time series of other mental states such as relaxation, resting, and vigilance, to name a few. Therefore, our results primarily represent the first step toward realization of the potential of this feature extraction strategy. Accordingly, future research that is devised with larger sample sizes along with more rigorous experimental settings and quantitative validation measures is necessary to derive an informed conclusion on its performance.

## Author contributions

SK carried out the proofs and analyses. HS conceived the experiments and supervised their progress. RY conducted the conversational experiment. As the head of Hiroshi Ishiguro Laboratories (HIL), HI oversees the entire activity of all research teams and themes, ensuring the soundness of all proposals, quality of results, and their validity. SK and HS contributed equally in preparation of this manuscript.

### Conflict of interest statement

The authors declare that the research was conducted in the absence of any commercial or financial relationships that could be construed as a potential conflict of interest.

## Figures and Tables

**Figure A1:**
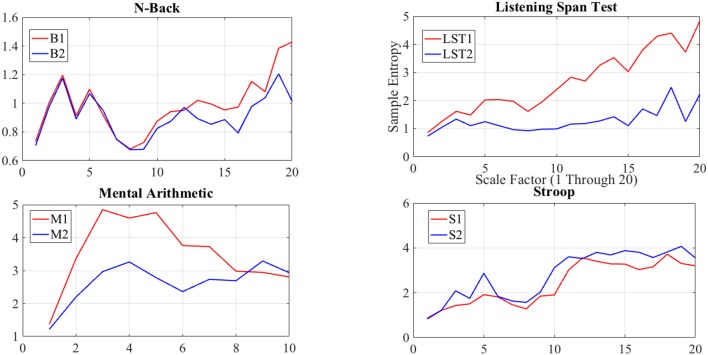
Grand averages of the mutli-scale entropy (MSE) values of the NIRS time series data of our participants. We use the pattern length *m* = 2, similarity criterion *r* = 0.15 and the range of 1 through 20 for the scale factor, as suggested by Goldberger et al. (2000).

## Footnotes

^1^Let *m*_*i*_ and *s*_*i*_ be the mean and slope of the *ith* segment. Then their combination, as it is practiced in NIRS analyses, implies the vector *v* = [*m*_*i*_, *s*_*i*_].

^2^Fano factor or coefficient of variation is a measure of dispersion of a probability distribution of a random process, *X*, that is expressed as $F_{X}=\frac{\sigma_{X}^{2}}{\mu_{X}}$ with $\sigma_{X}^{2}$ and μ_*X*_ representing the variance and mean of *X*, respectively.

## Appendix

### Multiscale entropy analysis of the WME

In our analyses, we found similar indication of non-significant difference between N-back subtasks through application of DE as well as mean of the NIRS time series of the brain activity of the participants. We further suggested that such a similarity is due to the significant resembling dynamics of these subtasks, and consequently the equivalence in their imposed cognitive loads on human subjects, as opposed to insensitivity of DE. In addition, we provided support for our claim from the comprehensive results on study and analysis of this N-Back WM task (Owen et al., 2005; Fishbum et al., 2014). In this Appendix, we present additional evidence in support of our claim through complexity analysis of the brain activity of our participants during N-Back WM task.

Referring to Figure A1, we observed non-significant difference in the complexity of the brain activity of the participants in response to B1 and B2 tasks, as illustrated through analysis of their respective multiscale entropy (MSE) (Costa et al., 2002, 2005). Whereas we observed significant difference in the level of complexity of the brain activity of the participants during LST [*p* < 0.001, *T*_(38)_ = 4.21, SD = 0.86], mental arithmetic [*p* < 0.03, *T*_(38)_ = 2.19, SD = 0.87] and Stroop [*p* < 0.03, *T*_(38)_ = −2.37, SD = 1.0], it was non-significant in case N-back WM tasks [*p* = 0.17, *T*_(38)_ = 1.41, SD = 0.18].
